# Gold a Temporary Stopping

**Published:** 1877-08

**Authors:** C. M. Wright

**Affiliations:** Basel, Switzerland


### GOLD A TEMPORARY STOPPING.

BY C. M. WRIGHT, D. D. S., BASEL, SWITZERLAND.

In the published transactions of the Mississippi Valley Dental Society, in the April number of the *Dental Register*, page 161, Dr. H. A. Smith, of Cincinnati, a gentleman well and favorably known to the dental profession for many years, a gentleman distinguished for the deep interest he has taken in the advancement of the profession, and for an independent and careful judgment of professional matters, and an accomplished and cultivated operator, makes the following reply to a question on the subject of "Dental Caries: Cause and Prevention": "The main reliance of the profession is filling. *In a vast number of cases it does not meet the requirement.* My observation for the last few years has led me to doubt somewhat the efficacy of mere filling for the prevention of caries. *A great many approximal fillings must be done over many times.* I have kept some statistics, and I think that forty per cent. of the fillings I make are approximate. * * * These bicuspid fillings do not always prevent decay. If they are put in at a period ranging from fourteen to twenty, they have to be done over in a *majority* of *cases.* We promise too much, etc." The following speaker so far accepted these remarks, that he said: "Any one who has been in practice ten years has seen this very thing." I do not quote these opinions for the purpose of disagreeing with them, but for exactly the contrary reason. If they are true in America, they are true here on the continent, too, as far as my observation and experience teach me. And yet hour after hour, day after day, we stand or sit, bent over these holes in the human teeth, and sweat and carve and file and chisel and pack gold, and a majority of forty cent. of our work is temporary. What modest men dentists should be! How humbly they should kneel only to kiss the hem of the garment of Science! How the bowed head and bent back assumed during their daily labor should be made an emblem of their hearts? And yet, how our memories are loaded with the blatant boastings of our brothers! How our ears tingle with the mouthing self-praise of so many of our confreres!

I have felt deeply and often the truth of these remarks above quoted, and have been stimulated to extra pains, to more persistent patience, to more determined efforts, in the struugle for excellence and perfection—and I meet a brother with half my physical vitality, half. my experience, who tells me of his grand operations and of their permanence, and in a fatherly way advises me to emulate him. Then, in the still hours of the night, as I lay and meditate on the subject, I think, "Well, if this man is honest and truthful, Nature has denied me the talents necessary to a dentist. Oh! that I could find some other *congenial* employment, in which bread and butter, good clothes, comfortable lodgings, and a little of the cakes and ale of life could be honestly earned." And sometimes, as I feel that I am too old to begin all over in some other profession, I even wish that I could occupy the position of heir to a great estate. Morning comes and labor begins, and—yes, truly! it is a patient of this talented operator—but alas! the fine operations do not appear. The crown cavities and the fillings in accessible places have been treated in a masterly manner, but majority of the *forty per cent.* cases are—well, are the cases that *I* must attend to at this sitting. In this little city I have fallen heir to a practice which has been held successively by four excellent dentists for seventeen or eighteen years. One of the four, the first—the lamented Dr. S. C. Putnam, of New York—has passed away, and the other three are still holding first-class positions as operators and dentists in the ranks of the profession, in different cities on the continent.

Daily the operations of the past stare me in the face, and the custom of my predecessors, of giving diagram records to their patients, many of whom present them to me for examination, has been of great interest and advantage to me. Here is a bicuspid, for instance, upon which Nos. 1, 2 and 3 have operated, and which No. 4 has extracted. Here is a six-year molar; No. 1 has left a beautiful crown operation, Nos. 2, 3 and 4 have left their marks, and the present incumbent is curing an abscess, or trying to—and so on. Is this not enough to make us modest? These were not common operators, but first-class, conscientious and capable dentists, and their work was for the best class of society. Given good quality of teeth, and these operations would, in a vast majority of cases, present what might be called splendid operations. Given the fact that the teeth of this neighborhood are as soft and chalky and irregular, and as liable to the ravages of caries of all the various types, as are the teeth in America, and we have the melancholy truth enunciated by Dr. Smith proved again. "What shall we do to be saved?" is an old question; and on one occasion, when the preacher had nearly exhausted all the inflexions in his elocution in playing upon this question, an old tar who happened to be one of the congregation profanely called out in answer, "Eat salt, d——n you!" But a real authority on the chemistry of the mouth stated, at this same meeting of the Mississppi Valley Association, that salt will not save the teeth; that the tendency of the white and brown variety of caries is greatly increased by the use of salt; that an alkaline condition of the fluids of the mouth is also said not to be a safe condition for the teeth; that eggs, lean meats, onions, etc., when the black variety exists, should be avoided, etc. But what can we, as dentists, do? We cannot direct the diet of patients any easier than can the family physician. We are compelled to fight with excavators and pluggers. We are compelled to be mechanics. We are compelled to acknowledge the superior power of the enemy, and simply watch and pray and stop the little encroachments as well as we can; and when finally, the battle is won by the enemy, we must have a convenient conscience, and smilingly acknowledge defeat, but have confidence in the good intentions of our profession and of our own labors.

There is in Paris an American dentist, who, it is said, by his agreeable manners and an aristocratic title, has such perfect control over his patients, that they believe that the teeth that are this year filled with gold, may next year be filled with gutta-percha, and the next year, again, be extracted, and replaced by porcelain ones. I heard a half dozen dentists laughing at this idea last summer in Paris, as they sat in the court of the Grand Hotel, sipping cool drinks and smoking Havanas; and I must acknowledge that I laughed too. It was a novel idea. But later reflection has taken the joke all out of it, and I have seriously asked if this dentist is not wiser than his generation? Would it not be better if patients were taught to believe that our operations are only temporary; that disease is stronger than our skill; that only constant vigilance can avail at all; and that while we stay the hand of the destroyer by our skill, he will in all probability beat us in the end? Then when we do save for a life-time an entire denture composed of delicate teeth, what credit we should receive! No other learned profession claims to do as much as we do in our line. The physician cannot tell surely the length of life a sick man may have. The lawyer only agrees to do the best he can toward success for his client. The professor cannot make his students absorb knowledge, and would not think of guaranteeing to do so. The architect—among the mechanical professions, it is true—is held responsible if his house or bridge falls down; but not if it is struck by lightning or falls in an earthquake. The dentist, however, meets the disapproving looks of his patient if a filling in a bicuspid fails, and yet "they have to be done over in a majority of cases."

Professor Taft, of Cincinnati, once said, "I don't want to hear of men's failures; we all have enough of them. I want to hear of their successes, etc." But as this was some ten or twelve years ago, perhaps it would be good to have a good season of failures—of acknowledged failures—and perhaps after awhile the question of "What may our patients reasonably expect from our services?" will be so thoroughly set at rest, that our *status* can be assured. Perhaps we shall have the courage to teach our patients that caries and disease have not yet been conquered by science and skill; but that with their co-operation, and by quarterly or monthly examinations by the dentist, even bad cases may be conquered *for a time*, and splendid teeth may be kept good till old age comes, in spite of the luxuries of civilization. Perhaps we ourselves may learn that gold is a material used as a *temporary filling* by dentists, excepting in teeth of excellent quality and in favorable positions, when it *may be* considered permanent.—*Johnston's Dental Miscellany.*

